# Nonindustry-Sponsored Preclinical Studies on Statins Yield Greater Efficacy Estimates Than Industry-Sponsored Studies: A Meta-Analysis

**DOI:** 10.1371/journal.pbio.1001770

**Published:** 2014-01-21

**Authors:** David Krauth, Andrew Anglemyer, Rose Philipps, Lisa Bero

**Affiliations:** 1 Department of Clinical Pharmacy, University of California San Francisco, San Francisco, California, United States of America; 2 Institute for Health Policy Studies, University of California San Francisco, San Francisco, California, United States of America; University of Edinburgh, United Kingdom

## Abstract

Industry-sponsored clinical drug studies are associated with publication of outcomes that favor the sponsor, even when controlling for potential bias in the methods used. However, the influence of sponsorship bias has not been examined in preclinical animal studies. We performed a meta-analysis of preclinical statin studies to determine whether industry sponsorship is associated with either increased effect sizes of efficacy outcomes and/or risks of bias in a cohort of published preclinical statin studies. We searched Medline (January 1966–April 2012) and identified 63 studies evaluating the effects of statins on atherosclerosis outcomes in animals. Two coders independently extracted study design criteria aimed at reducing bias, results for all relevant outcomes, sponsorship source, and investigator financial ties. The I^2^ statistic was used to examine heterogeneity. We calculated the standardized mean difference (SMD) for each outcome and pooled data across studies to estimate the pooled average SMD using random effects models. In *a priori* subgroup analyses, we assessed statin efficacy by outcome measured, sponsorship source, presence or absence of financial conflict information, use of an optimal time window for outcome assessment, accounting for all animals, inclusion criteria, blinding, and randomization. The effect of statins was significantly larger for studies sponsored by nonindustry sources (−1.99; 95% CI −2.68, −1.31) versus studies sponsored by industry (−0.73; 95% CI −1.00, −0.47) (*p* value<0.001). Statin efficacy did not differ by disclosure of financial conflict information, use of an optimal time window for outcome assessment, accounting for all animals, inclusion criteria, blinding, and randomization. Possible reasons for the differences between nonindustry- and industry-sponsored studies, such as selective reporting of outcomes, require further study.

## Abstract 2

This study by Lisa Bero and colleagues uses published preclinical statin research to show that nonindustry-funded animal studies yield more efficacious drug results than do industry-funded ones.

## Author Summary

Industry-sponsored clinical drug studies are associated with publication of outcomes that favor the sponsor, even when controlling for potential bias in the methods used. However, the influence of sponsorship bias has not been examined in preclinical animal studies. We performed a meta-analysis to identify whether industry sponsorship is associated with increased risks of bias or effect sizes of outcomes in a cohort of published preclinical studies of the effects of statins on outcomes related to atherosclerosis. We found that in contrast to clinical studies, the effect of statins was significantly larger for studies sponsored by nonindustry sources versus studies sponsored by industry. Furthermore, statin efficacy did not differ with respect to disclosure of financial conflict information, use of an optimal time window for outcome assessment, accounting for all animals, inclusion criteria, blinding, and randomization. Possible reasons for the differences between nonindustry- and industry-sponsored studies, such as selective outcome reporting, require further study. Overall, our findings provide empirical evidence regarding the impact of funding and other methodological criteria on research outcomes.

## Introduction

Preclinical studies are performed to evaluate a drug's efficacy in animal models [1]. In addition, the results from animal studies are a critical—and often the only—input to evaluating potential toxicity of drugs before they proceed to human testing. Minimizing bias in the design, conduct, and reporting of preclinical animal research should produce more methodologically sound studies and results that provide better protection of humans from exposure to drugs tested in toxicology studies. Policies to reduce biases in preclinical animal studies should lead to the initiation of appropriate clinical trials that are an efficient use of resources and minimize risk to humans.

For the evaluation of human clinical research, there is a distinction between assessing risks of bias and methodological quality. Risks of bias are methodological criteria of a study that can introduce a systematic error in the magnitude or direction of the results [2]. Some risks of bias in animal studies have been empirically identified. For example, analyses of animal studies testing interventions for stroke, multiple sclerosis, and emergency medicine have shown that lack of randomization, blinding of investigators, specification of inclusion and exclusion criteria for the animal subjects, statistical power, and failure to use comorbid animals are associated with inflated effect estimates of pharmaceutical interventions [3]–[7]. Using an optimal time window for outcome assessment and specifying whether all animals in the study are accounted for are also associated with reduced risks of bias [8],[9]. An assessment of a study's methodology also includes evaluation of additional study criteria related to how a study is conducted (e.g., in compliance with animal subjects guidelines) or reported (e.g., study population described).

Considerable evidence shows a strong association between industry sponsorship, investigator financial conflicts of interest (COIs), and biased outcomes in clinical research [2]. Industry sponsorship biases the written research record towards outcomes that are favorable to the sponsor, even when controlling for study design criteria [10]–[16]. Financial ties between clinical researchers and industry have also been associated with reduced data sharing [17]–[19]. There is little evidence regarding the influence of these financial COIs on the integrity of preclinical animal studies.

Statins are an interesting class of drugs for investigating biases that influence research outcomes because there are a number of statins manufactured by competing companies; thus, there are financial incentives to develop statins that are more effective or safer than others. Previous research has shown that randomized controlled clinical trials of head-to-head comparisons of statins with other drugs are more likely to report results and conclusions favoring the sponsor's product compared to the comparator drug, even when controlling for other risks of bias [14]. Clinically, statins are widely prescribed as effective agents for lowering cholesterol and other lipids. Statins have a number of other potential clinical uses that have been tested in animals including treating atherosclerosis, fracture healing, and endothelial dysfunction, and statins possess both anticarcinogenic and anticoagulant effects [20],[21].

The objective of this study is to determine whether industry sponsorship is associated with increased effect sizes of efficacy outcomes and/or increased risks of bias in a cohort of published animal studies examining the effects of statins on atherosclerosis. We show that nonindustry-sponsored animal studies contained outcomes that were more favorable to the test statin compared to industry-sponsored studies and that statin efficacy did not differ between studies with high and low risks of bias.

## Results

**Figure 1:**
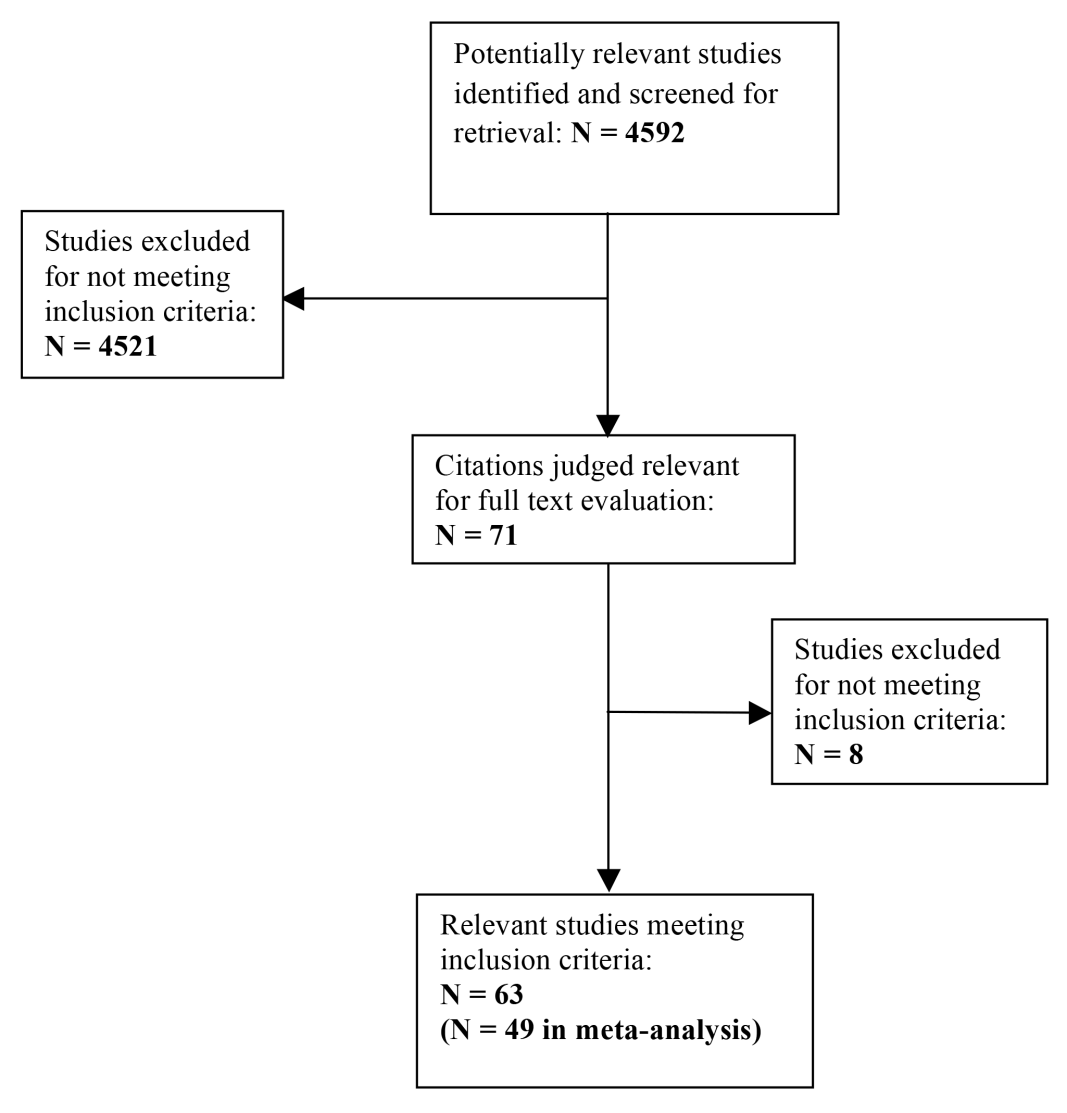
Flow of included studies. N indicates the number of studies.

As shown in Figure 1, we identified 4,592 potentially relevant studies. After screening study titles and abstracts, 71 citations containing atherosclerosis outcomes met our inclusion criteria and were identified for full text evaluation. After reviewing the full text, eight studies did not meet our inclusion criteria and were excluded. Overall, 63 total articles were included in this study. Of these 63 articles, 54 had quantitative data and 49 had analyzable data for the meta-analysis.

Table 1 shows the frequencies of the study design criteria of included studies by sponsorship source. Only three of the 63 included studies were sponsored solely by industry sources, although 16 were partially sponsored by industry. The majority of studies (35 of 54 studies; 64.8%) that reported quantitative results reported results that favored statins. Similarly, 52 of 63 studies contained author conclusions that favored statin use.

**Table 1:** Characteristics of included studies by sponsorship source (*n* = 63).

			Sponsorship Source			
Characteristic	Category	Total	Any Industry^1^ (*N* = 19)	Nonindustry(*N* = 28)	No Disclosure (*N* = 15)	No Sponsor (*N* = 1)
Comparison Group	Statin versus nonstatin drug	33	9	17	6	1
	Statin versus placebo	30	10	11	9	0
Sample Size	Range	9–138	20–138	9–120	12 to 50	36
Outcome Assessment	Laboratory analysis	61	18	28	14	1
	Mortality	2	1	0	1	0
Risk of Bias	Randomization	30	7	20	3	0
	Concealment of allocation	0	0	0	0	0
	Blinding	22	10	11	1	0
	Inclusion/exclusion criteria	3	2	0	1	0
	Sample size Calculation	0	0	0	0	0
	Test animal description	63	19	28	15	1
	Animal environment described	61	19	27	14	1
	Dose/response model	30	9	11	10	0
	Optimal time window investigated	8	2	3	3	0
	All animals accounted for	39	9	18	11	1
	Intention-to-treat analysis	0	0	0	0	0
Results^2^	Favors statin	35	9	18	8	0
	Does not favor statin	10	4	3	3	0
	Neutral	9	4	4	1	0
Conclusion	Favors statin	52	18	21	13	0
	Does not favor statin	3	0	2	1	0
	Neutral	8	1	5	1	1

^1^ The “any industry” category includes three studies sponsored solely by industry and 16 sponsored by industry and nonindustry sources.

^2^ Includes 54 studies that reported quantitative results; *n* = 9 did not report quantitative results.

The most commonly researched statin was simvastatin, evaluated in 31% (15 of 49) of studies, followed by pravastatin (24%, 12/49), and atorvastatin (20%, 10/49). Other statins studied included fluvastatin (12%, 6/49), lovastatin or rosuvastatin (8%, 4/49), and cerivastatin or pitavastatin (6%, 3/49). Of the 63 studies, five studies [22]–[26] evaluated multiple statins, of which four were included in the meta-analysis [22]–[24],[26].

Overall, test animal characteristics and a description of the animal environment were the most commonly reported criteria [62 of 63 studies (98.4%) and 60 of 63 studies (95.2%), respectively].

### Specific Measures by Category of Outcome

The results were classified into the following categories, many of which contained multiple measures: (1) vessel measure, (2) plaque measure, (3) incidence of lesions, (4) occlusion, (5) plaque type/severity, (6) coronary stenosis, and (7) plaque stability.

Vessel measures included a myriad of outcomes: aortic lesion development, intima/media ratios, intimal thickening, maximal thickness, vessel wall area, lumen area, media size, carotid plaque, thickness of elastic layer, external diameter, aortic peak velocity (Vp), mean velocity (Vm), velocity time integral (VTI), and the external elastic membrane area (EEMA). Plaque measures included atherosclerosis plaque/lesion/surface area/thickness, plaque volume, aorta/artery maximum thickness, aorta/artery plaque volume, perimeter of plaque, average size of individual lesions, fatty streak area, frequency of lesions with hemorrhage, necrotic area, area of atheroma, and large necrotic core. Lastly, other measures included occlusion and fibrous cap measures.

The most commonly reported outcome categories were plaque measures, which were reported in 50 of 63 studies (79.4%), and vessel measures, which were reported in 26 of 63 studies (41.3%). Six studies (9.5%) reported coronary stenosis outcomes, whereas five studies (7.9%) reported plaque stability outcomes. Other outcome categories reported in identified studies were plaque type/severity (*n* = 4; 6.3%), incidence of lesions (*n* = 2; 3.2%), and occlusion outcomes (*n* = 1; 1.6%).

### Animal Species Studied

Various rabbit species were studied in 55.6% (35 of 63) of studies and mice in 31.7% (20 of 63) of studies. Other species studied included hamsters (*n* = 2), monkeys (*n* = 2), rats (*n* = 2), chickens (*n* = 1), and guinea pigs (*n* = 1).

### Financial COIs

Overall, only 1.6% (1 of 63) of studies disclosed a financial COI of authors, 15.9% (10 of 63) of studies stated that all authors had no COIs to report, and 82.5% (52 of 63) of studies did not have any COI disclosure statement.

### Role of the Financial Sponsor

Among the 47 studies with a disclosed sponsor of any type, 45 studies (95.7%) did not mention whether the sponsor was involved in the study, one study (2.1%) explicitly stated that the sponsor was not involved in the study, and one study (2.1%) stated that the sponsor was involved in the study.

### Results and Conclusions Reported

#### Favorable results

Among studies with quantitative results and any disclosed sponsorship (*n* = 42), nearly half of studies with some industry sponsorship reported favorable results (*n* = 9 of 19 studies; 47%), whereas 72% (*n* = 18 of 25 studies) of nonindustry-sponsored studies reported favorable results. The relative risk of having favorable results when comparing industry-sponsored studies to studies without industry sponsorship was RR = 0.74 (95% CI 0.44–1.23).

#### Favorable conclusions

Studies with some industry sponsorship were more likely to have conclusions that favored the statin (18 of 19 studies, 94.7%) than studies sponsored by nonindustry sources (21 of 28 studies, 75.0%). The relative risk of having favorable conclusions when comparing industry-sponsored studies to studies without industry sponsorship was RR = 1.26 (95% CI 0.99–1.60).

### Meta-Analysis

**Figure 2:**
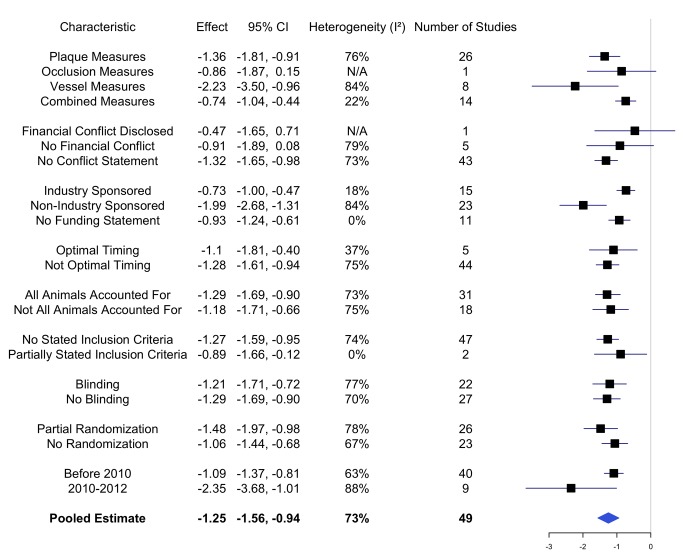
Stratified meta-analysis of 49 animal studies estimating effect of statins on atherosclerosis risk. Horizontal lines indicate 95% confidence intervals (CIs) and squares reflect the point estimate. The blue diamond reflects the pooled estimate across all studies and the vertical line reflects the null hypothesis.

As shown in Figure 2, across all studies with analyzable results (49 studies with 184 outcomes measured in 954 animals), the pooled effect significantly favors statin use for atherosclerosis-related efficacy outcomes (−1.25; 95% CI −1.56, −0.94), with substantial heterogeneity (I^2^ = 73%). Ninety-four percent (*n* = 46 of 49) of average effects yielded point estimates favoring statins, whereas 6% of average effects yielded point estimates favoring controls.

### *A Priori* Subgroup Analyses

#### Sponsorship source

As shown in Figure 2, the effect of statins is greater in studies with nonindustry sponsorship versus industry-sponsored studies (test for subgroup differences: *p* value<0.001) and in studies with nonindustry sponsorship versus studies with no sponsorship statement (test for subgroup differences: *p* value = 0.006). The effect of statins does not differ between studies with industry sponsorship and studies with no sponsorship statement (test for subgroup differences: *p* value = 0.36).

Using the 49 studies with analyzable results, we also conducted a sensitivity analysis using more specific sponsorship groupings: industry sponsored (*n* = 3), combined industry and nonindustry sponsored (*n* = 12), nonindustry sponsored (*n* = 23), and no funding statement (*n* = 11). This analysis yielded similar results between studies with industry sponsorship alone and studies with both industry and nonindustry sponsorship (industry sponsorship alone: 0.73 95% CI −1.00, −0.47; I^2^ = 18%; and industry and nonindustry sponsorship: 0.67 95% CI −1.00, −0.35; I^2^ = 29% versus nonindustry sponsorship: −1.99 95% CI −2.68, −1.31; I^2^ = 84%; tests for subgroup differences: *p* value<0.001). Furthermore, grouping studies with no financial statement with industry-sponsored studies also yielded similar results (industry sponsorship: 0.81 95% CI −1.01, −0.60; I^2^ = 111% versus nonindustry sponsorship: −1.99 95% CI −2.68, −1.31; I^2^ = 84%; test for subgroup differences: *p* value<0.001).

#### Outcome measures

As shown in Figure 2, the effect of statins is greater in studies with only plaque or vessel measures versus combined measures (tests for subgroup differences: *p* value = 0.03).

#### Year of publication

The studies were published between 1991 and 2012. As shown in Figure 2, studies published between 2010 and 2012 yielded greater efficacy estimates than studies published before 2010 (*p* = 0.07). Although we hypothesized that more recent studies would have lower risks of bias given that the Animal Research: Reporting In Vivo Experiment (ARRIVE) Guidelines for reporting animal research were introduced in 2010, we found that only randomization, whether all animals were accounted for, and sample size were reported more frequently after publication of the ARRIVE Guidelines. Randomization was reported in 11 of the 15 (73.3%) studies that were published post-ARRIVE compared to 19 of the 48 (39.6%) studies published pre-ARRIVE. All animals were accounted for in 12 of the 15 (80%) studies published post-ARRIVE compared to 27 of the 48 (56.3%) studies published pre-ARRIVE. Sample size was reported in all 15 studies published post-ARRIVE compared to 41 of 48 studies (85.4%) published pre-ARRIVE.

As shown in Figure 2, studies with financial COI disclosures, optimal timing of outcome measures, all animals accounted for, stated inclusion criteria, blinding, or randomization did not show greater efficacy of statins compared to studies without these criteria. In only one study did an author disclose a financial COI; therefore, subgroup analysis by this criterion was not performed. Instead, we conducted a subgroup analysis by whether the study contained a COI statement or not. The effect of statins is not different in studies with no financial conflict compared to studies with no conflict statement (test for subgroup differences: *p* value = 0.44). The effect of statins is not different in studies with optimal timing of outcome measurement compared to studies without optimal timing (test for subgroup differences: *p* value = 0.66), in studies with all animals accounted for compared to studies that did not account for all animals (*p* value = 0.74), in studies with no inclusion criteria described compared to studies with partial inclusion criteria (*p* value = 0.37), or in studies with blinding compared to studies with no blinding (*p* value = 0.81). No studies had full randomization. The effect of statins is not different in studies with partial randomization compared to studies with no randomization (*p* value = 0.19).

### Risks of Bias by Sources of Sponsorship

To explore the differences in risks of bias between studies reporting different sources of sponsorship, we compared pooled efficacy estimates from studies with sponsorship from industry, nonindustry source(s), or no sponsorship statement stratified by risks of bias that are associated with effect size: randomization (partial versus no randomization), blinding of investigators (yes versus no), and all animals accounted for (yes versus no).

Comparisons between nonindustry-sponsored studies and studies sponsored by industry sources yielded higher efficacy estimates for statins in nonindustry-sponsored studies with partial randomization (test for subgroup difference: *p* = 0.008). Similarly, comparison between nonindustry studies with no randomization and studies sponsored by industry sources with no randomization yielded higher efficacy estimates for statins in nonindustry studies (test for subgroup difference: *p* = 0.06).

Comparisons between industry-sponsored studies and nonindustry-sponsored studies remained significantly different, yielding higher efficacy estimates for statins in nonindustry-sponsored studies with blinding (test for subgroup difference: *p* = 0.002).

Comparisons between industry-sponsored studies and nonindustry-sponsored studies remained significantly different, yielding higher efficacy estimates for statins in nonindustry-sponsored studies with all animals accounted for (test for subgroup difference: *p* = 0.001).

### Sensitivity Analysis Using Fixed Effect Model

We performed a sensitivity analysis using a fixed-effects model rather than a random-effects model to evaluate differences in pooled estimates. The pooled effect of statins on the risk of atherosclerosis using a fixed-effects model was more attenuated (SMD = −1.01; 95% CI −1.17, −0.86) than the random-effects model (SMD = −1.25; 95% CI −1.56, −0.94). Similarly, the pooled effect among nonindustry-sponsored studies using a fixed-effects model was less (SMD = −1.44; 95% CI −1.70, −1.17) than the random-effects model (SMD = −1.99; 95% CI −2.68, −1.31). The pooled effect among industry-sponsored studies remained nearly unchanged (SMD = −0.72; 95% CI −0.96, −0.48 versus SMD = −0.73; 95% CI −1.00, −0.47). Tests of significance comparing subgroups remained significant in fixed-effects models.

## Discussion

Nonindustry-sponsored studies yielded greater efficacy estimates than industry-sponsored studies. Despite subgroup analyses by risks of bias, including financial COI information, optimal timing of outcome measurement, accounting for all animals, inclusion criteria, blinding, and randomization, efficacy estimates for nonindustry-sponsored studies remained significantly greater than industry-sponsored studies. This finding does not correspond to clinical studies that have examined sponsorship bias. Reviews of clinical drug studies have shown that industry funding sources and financial ties of investigators (including university- or industry-affiliated investigators) are associated with increased treatment effect sizes and other favorable outcomes for the sponsors [27]. One reason for the discrepancy between the association of funding source and outcome in preclinical and clinical studies could be that the interests of the pharmaceutical industry are best served by underestimating efficacy prior to clinical trials and overestimating efficacy in clinical trials. By underestimating efficacy in preclinical studies, the pharmaceutical industry could reduce the money spent on clinical trials that did not lead to marketable products. By overestimating efficacy in clinical trials, the pharmaceutical industry could skew the evidence towards findings that would lead to drug approval and a marketable product.

Another possible explanation for our finding that nonindustry-sponsored studies demonstrated greater efficacy than industry-sponsored studies, independent of other risks of bias, is that an overwhelming number of studies in our sample had statistically significant results in favor of the test statin. Ninety-four percent (*n* = 46 of 49) of studies yielded point estimates favoring statins. This finding is consistent with research demonstrating that the vast majority of clinical studies report statistically significant results for the interventions [28]. Similarly, overly optimistic findings in animal studies could skew the data and send potentially ineffective drugs to clinical trials. Several studies have shown that favorable findings in animal studies often are not reproduced in clinical trials [29]–[32].

Among clinical studies, most discovered true associations are inflated due to methodological flaws [28]. Although we could not detect differences in risks of bias between the nonindustry- and industry-sponsored studies in our sample, substantial risks of bias existed across all studies. For example, none of the studies in our sample included sample size calculations, conducted intention-to-treat analysis, or had concealment of allocation. Approximately half of the studies did not report randomization, blinding of investigators, or the inclusion and exclusion criteria for the animal subjects.

Our inability to detect an association of risks of bias with effect sizes might be due to the poor reporting of these criteria. Among the 36 journals where the 63 studies were published, we determined that eight journals (22.2%) required only that authors report compliance with animal welfare regulations in their article, 17 (47.2%) had specific reporting requirements, and 11 (30.6%) did not have any reporting requirements. Recent calls for reporting criteria for animal studies recognize the need for the adoption and enforcement of journal reporting standards [33],[34]. In clinical research, reporting of risk of bias criteria improved as investigators began performing risk of bias assessments for systematic reviews and other purposes and journals began adopting reporting standards [35]. As happened for clinical research, reporting of animal research is also likely to improve if risk of bias assessments becomes more common.

Reporting biases (i.e., failure to publish entire studies or selective outcome reporting) could explain the skewing of our sample towards favorable results and the very small proportion of industry-sponsored studies identified. The effect of publication bias has been documented in both animal and clinical studies. Studies of reporting biases in clinical research have shown that industry-sponsored clinical trials are less likely to be published in full than studies with other sponsors [36]–[40]. Similarly, a meta-analysis of animal studies assessing interventions for stroke showed that publication bias could account for at least a third of the efficacy reported in systematic reviews of animal stroke studies [6]. Furthermore, data from a national survey of all animal laboratories in the Netherlands indicate that publication bias appears to be more prevalent in industry-sponsored animal research relative to animal research sponsored by nonindustry sources [41].

Our analysis of the direction of outcomes showed that industry-sponsored studies were less likely to have results that favored the statin, but more likely to have conclusions favoring the statin, compared to studies sponsored by nonindustry sources. The greater discordance between results and conclusions in industry-sponsored studies compared to nonindustry-sponsored studies has also been observed in meta-analyses of randomized controlled trials and trials of drugs conducted in human [16],[27]. The presence of “spin” in the conclusions of randomized trials—that is, specific reporting strategies to highlight that the experimental treatment is effective despite a statistically nonsignificant effect for the primary outcome—has been demonstrated [42]. Further analysis of spin in the conclusions of animal studies is warranted.

Our study reveals a lack of reporting of harm-related metrics. Not a single study in our cohort assessed adverse events following the statin intervention; all 63 studies were nonequivalence efficacy studies. As toxicity data from animal studies must be submitted to drug regulatory authorities before a compound can proceed to testing in humans, it is surprising that so little data on harm appear in the published scientific literature. Although only one out of 22 study criteria from the Consolidated Standards of Reporting Trials (CONSORT) checklist addresses reporting of drug harms, attempts have been made to improve reporting of harm data [43].

### Limitations

This study has a few limitations. We restricted our search to the Medline database. Although we may have retrieved further studies had we searched additional databases, we found a sufficient number of studies (*n* = 63) to test our hypothesis examining the association of industry sponsorship, risks of bias, and research outcomes. Many of the studies we included had small samples sizes and measured multiple outcomes in a single animal. Therefore, when combining outcome measures within studies, we were unable to avoid double-counting animals within studies. We obviated this issue by averaging effect estimates and variances within studies. It should be noted that variances of SMDs are expected to be similar for different outcomes on the same animals. Therefore, the average variance across multiple outcomes should be similar to the variance for one outcome. Some outcomes were excluded from the meta-analyses due to missing data or the type of outcome estimates calculated. Thus, it is possible that our analyses overrepresented studies and outcomes with analyzable data.

Additionally, the types of statins compared between studies varied. Although the aim of this study was not to compare the efficacy of specific types of statins, we acknowledge the possibility of added heterogeneity due to unequal efficacy between statin types. Additional heterogeneity was likely present due to differences in or inadequate descriptions of the control groups in the studies.

### Conclusion

Nonindustry-sponsored studies demonstrated greater efficacy than industry-sponsored studies, independent of other risks of bias. Because demonstrating drug efficacy in human studies is linked to drug company profits, drug companies may have more incentive to publish favorable efficacy findings of human drug studies than animal studies. Overall, the influence of funding source, risks of bias (such as lack of randomization and blinding), overwhelming proportion of studies reporting statistically significant efficacy outcomes, and lack of reporting of harm outcomes suggest a need for improving the integrity of preclinical animal research.

## Materials and Methods

The selection criteria for studies, data extraction, and analyses were all determined *a priori*. A completed PRISMA checklist for this study can be found in Text S2.

We searched for all nonhuman animal (e.g., mice, rabbits, hamsters, monkeys, rooster, and chicken) studies of statins that compared a statin to a nonstatin comparator drug or placebo and measured outcomes for atherosclerosis. We identified studies testing the following statins: atorvastatin, cerivastatin, fluvastatin, lovastatin, mevastatin, pitavastatin, pravastatin, rosuvastatin, or simvastatin. We did not exclude any type of statins.

### Inclusion/Exclusion Criteria

We reviewed abstracts of all citations and retrieved studies based on the following inclusion criteria: (1) study conducted in animals; (2) original research, defined as a study that presented original data and did not specifically state that it was a review; (3) statin drug compared to either a nonstatin drug or placebo; (4) efficacy and/or harm outcomes measured; and (5) assessed effect of statin on at least one clinically relevant atherosclerosis-related outcome (including vessel measures, plaque measures, incidence of lesions, measurements of occlusion, plaque type/severity, coronary stenosis, and/or plaque stability).

Studies with the primary objective of assessing the effect of a combination of a statin and another drug were included if a comparison between a statin-only treatment group and the other drug was made.

Studies were excluded if they met any of the following criteria: (1) pharmacokinetic or pharmacodynamic studies; (2) editorials, letters to the editor, commentaries, abstracts, unpublished reports, systematic reviews, and meta-analyses; (3) studies only comparing different doses of one type of statin; (4) studies only comparing statins to a nondrug intervention (e.g., diet, exercise); (5) studies in which the statin was present in all the comparison groups; (6) in vitro analysis; and (7) studies with no comparison groups.

Initially, abstracts and study titles were reviewed, and only those studies meeting our inclusion criteria were further scrutinized by reading the full text. Any studies that did not clearly meet the criteria after review of the full text were discussed by two authors and a decision was made about inclusion.

### Search Strategy

We searched Medline from January 1966 to April 2012 using a search term combination developed with input from expert librarians. Our search strategy contained the following Medical Subject Heading (MeSH) terms, text words, and word variants:

(atorvastatin OR cerivastatin OR fluvastatin OR lovastatin OR mevastatin OR pitavastatin OR pravastatin OR rosuvastatin OR simvastatin OR hydroxymethylglutaryl-CoA reductase inhibitors) AND (animal* OR preclinical OR “pre-clinical” OR mice OR rats OR rabbits OR dog OR dogs OR monkey OR monkeys OR “animal experimentation”[MeSH Terms] OR “models, animal”[MeSH Terms] OR “invertebrates”[MeSH Terms] OR “Animals”[MH] OR “animal population groups”[MeSH Terms]) NOT (humans[mh] NOT animals[mh:noexp]) AND (health effect OR health effects OR toxic OR toxicity OR toxicities OR efficacy OR efficacies OR toxicology OR safety OR harm* OR drug effects[sh] OR therapeutic use[sh:noexp] OR adverse effects[sh] OR poisoning[sh] OR pharmacology[sh:noexp] OR chemically induced[sh]) AND eng[la] NOT review[pt] NOT systematic review* NOT meta-analysis[pt]

### Data Extraction

For single-coded data collection, the following characteristics were collected from each included study by a single coder (D.K.):

#### Study characteristics

Title of the study, month of publication, year of publication, and journal name.

#### Author characteristics

The affiliation(s) of the author(s) was obtained from the study by-line and classified into (1) industry, if all authors were employed by industry; (2) nonindustry, if no author was employed by industry; or (3) combined if at least one author was employed by industry and at least one author was not employed by industry. If a single author had affiliations with industry and nonindustry sources, the study was coded as “combined.”

#### Study design criteria

For each study, the following study design criteria were collected: (1) name of statin used in the study; (2) the comparison groups (e.g., comparator statin, active comparator nonstatin drug, or placebo); (3) animal species and strain used in the study; (4) number of control and treated animals at the start of the study; (5) type of study defined as a harm study, efficacy study, or both; and (6) whether the outcome data that we collected were the result of a laboratory analysis or a test to evaluate the effects of drug treatment on morbidity or mortality. We identified outcome data as “laboratory analysis” if an investigator measured the area of an atherosclerotic lesion, plaque thickness, intima/media thickness levels, or other related atherosclerosis outcomes in vivo. If an investigator recorded the occurrence of statin-induced morbidity (e.g., tumor progression, neurological damage, etc.) or mortality, we coded the analysis as “morbidity” or “mortality,” respectively.

### Double-Coded Data Collection

Two coders (D.K. and R.P.) extracted data on risks of bias, effect size measurements for statin efficacy/harm outcomes, study sponsorship source(s), investigator financial ties, and author conclusions. The two coders worked independently and any discrepancies were discussed and adjudicated with a third coder making the decision if the discrepancy could not be resolved. The data extraction coding book is included in Text S1 [44].

### Study Design Criteria to Assess Risks of Bias and other Methodological Criteria

We developed a set of core risk of bias criteria using findings from our published systematic review of assessment instruments for animal research [45]. Among the 30 instruments evaluated in that review, the most commonly included criteria are randomization (included in 25 of 30 instruments), investigator blinding (23 of 30), and sample size calculation (18 of 30) [45], each of which are included in this study. We also assessed several other empirically tested criteria that are known to influence research outcomes in animal research and/or are related to favorable results or conclusions in clinical drug studies [3],[7],[8],[27],[45]–[52].

Some criteria included in our list are not associated with bias. For example, a statement of compliance with animal welfare requirements is a reporting issue. We included this criterion in our study as it was the most commonly required reporting criterion by the 36 journals that published the 63 studies included in this analysis. Similarly, although a sample size calculation is not associated with bias—as bias is not the same as imprecision—we included this criterion, as it is an important characteristic to consider in evaluating an overall body of evidence [45].

All criteria were coded as (1) yes, if the criterion was met; (2) no, if the criterion was not met; and when applicable (3) partial, if the criterion was partially met. The following criteria were assessed for each publication:

#### Randomization

Was the treatment randomly allocated to animal subjects so that each subject has an equal likelihood of receiving the intervention? Randomization was coded as (1) yes, (2) no, and (3) partial.

#### Concealment of allocation

Were processes used to protect against selection bias by concealing from the investigators how treatment was allocated at the start of the study? Concealment of allocation was coded as (1) yes, (2) no, and (3) partial.

#### Blinding

Was the investigator(s) involved with performing the experiment, collecting data, and assessing the outcome of the experiment unaware of which subjects received the treatment and which did not? Blinding was coded as (1) yes, (2) no, and (3) partial.

#### Test animal description

Did the author(s) describe in detail the test animal characteristics including the animal species, strain, substrain, genetic background, age, supplier, sex, and weight. At least one of these characteristics must be present for this criterion to be met. Test animal description was coded as (1) yes, (2) no, and (3) partial.

#### Environmental parameters

Did the author(s) adequately describe the housing and husbandry, nutrition, water, temperature, and lighting conditions? At least one of these characteristics must be present for this criterion to be met. Environmental parameters were coded as (1) yes, (2) no, and (3) partial.

#### Inclusion/exclusion criteria

Were criteria used for including or excluding subjects specified? Inclusion/exclusion criteria were coded as (1) yes, (2) no, and (3) partial.

#### Dose/response model

Was an appropriate dose-response model used given the research question and disease being modeled? Dose/response model was coded as (1) yes and (2) no.

#### All animals accounted for

Did the investigator account for attrition bias by detailing when animals were removed from the study and for what reason they were removed? All animals accounted for was coded as (1) yes, (2) no, and (3) partial.

#### Intention-to-treat analysis

Did the author(s) perform an intention-to-treat analysis (ITT)? ITT was coded as (1) yes, (2) no, and (3) partial.

#### Optimal time window investigated

Did the investigator provide sufficient time to pass before assessing the outcome? The optimal time window used in animal research should reflect the time needed to see the outcome. Optimal time window investigated was coded as (1) yes, (2) no, and (3) partial.

#### Statement of compliance with animal welfare requirements

Did the author(s) state whether or not they complied with regulatory requirements for the handling and treatment of test animals? Statement of compliance with animal welfare requirements was coded as (1) yes or (2) no.

#### Sample size calculation

Did the authors perform a sample size calculation to justify the total number of animals used in the study? Sample size calculation was coded as (1) yes or (2) no.

### Coding of Results

Only results for atherosclerosis-related outcomes were recorded. If multiple time points were reported, we included all time points in the meta-analysis as to not assume a primary endpoint or arbitrarily assign an endpoint in the analysis. For each result we collected the raw data (often derived from tables, graphs, figures, etc.), measure of effect, confidence interval, measure of variability, *p* value, and statistical test used.

Each outcome was assigned to one of the following categories: (1) vessel measure, (2) plaque measure, (3) incidence of lesions, (4) occlusion, (5) plaque type/severity, (6) coronary stenosis, and (7) plaque stability.

Results were then categorized as (1) favorable, if the result was statistically significant (*p*<0.05) and in the direction of the statin being more efficacious or less harmful (in the case of adverse effects); (2) unfavorable, if the result was not statistically significant (*p*>0.05) or significant in the wrong direction (e.g., statin statistically more harmful than nonstatin treatment group); and (3) neutral, if the statin was significantly different in the direction favoring the statin against one control group (e.g., early control) but not significantly different compared to a second control group (e.g., late control).

If an outcome was measured over multiple time points or concentrations, it was categorized as (1) favorable if at least one measurement was in favor of the statin or (2) unfavorable if there were no measurements in favor of the statin.

For the meta-analysis, for each included result, we extracted data for mean outcome, standard deviation (SD) or standard error (SE), and the number of treated and untreated animals.

### Sponsorship Source

The source of sponsorship for each study was categorized as (1) any industry, (2) nonindustry, (3) no sponsorship statement, and (4) no sponsorship.

### Financial Ties of Authors

Information about financial ties was coded as (1) at least one author of the study reported having a financial COI, (2) all authors reported having no COIs, and (3) there was no disclosure statement.

### Role of the Financial Sponsor

For studies with a disclosed sponsor of any type, the role of the sponsor was categorized as (1) not mentioned, (2) statement that the sponsor was not involved, and (3) statement that the sponsor was involved. When applicable, we reported specifically how the sponsor was involved (e.g., in the design, analysis, or dissemination of the study).

### Author Conclusion of Included Study

For each published article, author conclusions were categorized as (1) favorable to the statin if the overall conclusion suggested that the statin is efficacious and safe. Conclusions were also favorable if the data empirically supported an author's hypothesis that the statin was efficacious and/or safe. For studies containing combination therapies (e.g., statin combined with another nonstatin drug), conclusions were coded as (1) favoring the statin if the author's hypothesis that the drugs have synergistic effects was supported; (2) unfavorable to the statin if the overall conclusion suggested that the statin is not efficacious or safe, less effective or safe relative to the active comparator, or if the author's hypothesis was not supported; and (3) neutral to the statin if the authors did not draw a conclusion regarding the statin or stated that the limitations of the study were so severe that the results were not valid. Conclusions were also neutral if the hypothesis or conclusion did not mention the statin or if the statin was considered comparable (in efficacy or toxicity) to the active comparator.

### Statistical Analysis

We report the frequencies of each study design criterion and the coding of the results and conclusions by sponsorship source.

To test our hypothesis that industry sponsorship is associated with increased effect sizes of efficacy outcomes and/or increased risks of bias, we conducted a meta-analysis of the studies that had analyzable data. For a study to have analyzable data, an author needed to report both a mean value and a measure of dispersion (standard error or standard deviation) or provide adequate data so that we could calculate these measures ourselves. Not all studies containing quantitative (numerical) data had analyzable data.

We calculated the effect of statins using an SMD for each outcome. Due to the lack of independence of animals between outcomes within studies, we averaged SMDs and variances across outcomes for each study, yielding *k* average SMDs and variances for *k* studies. We pooled the data across studies and estimated summary average SMDs using random-effects models [53]. Specifically, we estimated the average SMD for each included study and used the inverse variance method to calculate study weights. The inverse variance method assumes that the variance for each study is inversely proportional to its importance; therefore, more weight is given to studies with less variance than studies with greater variance. The SMD null hypothesis (Ho: estimate = 0) states that there is no difference in effect of statin use on risk of atherosclerosis when compared to a control/placebo. A number less than zero suggests that the statin reduces the risk of atherosclerosis when compared to control or placebo. A number greater than zero suggests that the statin increases the risk of atherosclerosis when compared to the control or placebo.

We examined heterogeneity among the studies using the I^2^ statistic. We interpreted an I^2^ estimate greater than 50% as indicating moderate or high levels of heterogeneity. We anticipated high levels of heterogeneity as previous meta-analyses of animal studies have found high levels of heterogeneity between studies, potentially resulting from typical, small sample sizes in animal models [54].

We further investigated the potential causes of heterogeneity by conducting *a priori* subgroup analyses using the χ^2^ statistic with a significance level of 0.10. We conducted a subgroup analysis by type of outcome measure because we hypothesized that the efficacy of statins could vary by outcome. We performed subgroup analyses by study criteria that we hypothesized would be associated with effect sizes: financial ties of authors, sponsorship source, optimal timing, accounting for all animals, inclusion criteria, blinding, and randomization. We also performed a subgroup analysis by date of publication as we hypothesized that more recent studies would have lower risks of bias given that the ARRIVE Guidelines for reporting animal research were introduced in 2010. We assessed whether or not authors reported the following criteria more frequently postpublication of the ARRIVE Guidelines: a statement by authors regarding the ethical treatment of animals, a description of the test animal characteristics, environmental parameters, sample size, randomization, blinding of investigators, all animals accounted for, and inclusion/exclusion criteria.

We evaluated differences in pooled effect estimates between declared sponsorship sources by risk of bias criteria to determine if the effect between sponsorship sources differed by specific risks of bias.

## Supporting Information

Text S1 **The data extraction coding book is included in Text S1.**
(PDF)

Text S2 **A completed PRISMA checklist for this study.**
(DOC)

## Abbreviations

ARRIVE: Animal Research: Reporting of In Vivo Experiments
CI: confidence interval
COI: conflict of interest
CONSORT: Consolidated Standards of Reporting Trials
MeSH: Medical Subject Heading
SMD: standardized mean difference
