# Supplementary material for: Nonindustry-Sponsored Preclinical Studies on Statins Yield Greater Efficacy Estimates Than Industry-Sponsored Studies: A Meta-Analysis
Source: PLoS Biol. 2014 Jan 21;12(1):e1001770. doi: 10.1371/journal.pbio.1001770 (PMC3897361; doi:10.1371/journal.pbio.1001770)
Supplement: Text S1 — The data extraction coding book is included in Text S1. (PDF) [file pbio.1001770.s001.pdf]

STATINS

Coding Manual for Risks of Bias, other Methodological Criteria, and Research Outcomes

Instructions for Reviewers

Objective
The purpose of this project is to investigate whether industry research sponsorship and / or principal investigator financial ties are associated with methodological biases or biased research outcomes in a cohort of preclinical statin studies.

Instructions for Coding
For each study, it is recommended that the reviewer read through the entire research article and abstract before coding. Complete every question using the excel form provided. Please use the "comments" section to write any questions or concerns regarding a specific item. Also, please highlight or mark-up the paper you are coding so it will be easy to determine why a criterion was coded a particular way.

Part I. Study Background/Introduction/Journal Information (single coded)

1. Journal Characteristics
State the following:
(a) Title of the research study
(b) Month of publication
(c) Year of publication
(d) Journal name

2. Study Characteristics
(a) Name the statin(s) used in the study.

(b) Name the comparison groups that are tested (e.g. statin vs placebo OR statin versus active comparator).

(c) Name the animal species used in the study. For laboratory mice models, state what strain of mouse was used (e.g. Wistar rat, Sprague Dawley rat, Zucker rat, knockout mice, other).

(d) State the final sample size analyzed, including the sample size for each intervention group.

(e) Mark which type of study was conducted. If the study tested both the toxicity of using a statin and the efficacy of using the statin, mark both types.

3. Outcome Assessment
(a) State only those outcome names related to atherosclerosis. If the paper contains multiple outcomes, record only those outcomes related to atherosclerosis.

(b) Describe whether or not the outcomes are the results of a laboratory analysis or if the author reports morbidity or mortality of the animal subject as the endpoint. For example, if an investigator measured triglyceride levels, high density lipoprotein, low-density lipoprotein (LDL) levels in vivo, mark *"laboratory analysis"*. If an investigator recorded the occurrence of statin-induced morbidity (eg, tumor progression, neurological damage, etc.) or mortality, mark the outcome as *"morbidity"* or *"mortality"*, respectively.

Part II. Risks of Bias and Other Methodological Criteria (double coded)

4. Randomization - *Was the treatment randomly allocated to animal subjects so that each subject has an equal likelihood of receiving the intervention?*

Check "*Yes*" if the investigator provided a detailed description of the randomization process. If an author says that randomization methods were used to allocate treatment to test subjects, but does not describe the randomization process, mark *"Partial"*. If the investigator does not explicitly state that randomization was used to allocate treatment to animal subject, mark *"No"*. Regardless of whether or not the author stated that randomization was used, if the author described a process whereby each subject did not have an equal likelihood of receiving the intervention (for example, "alternate allocation" where every second animal receives the intervention), mark *"No"*.

5. Concealment of Allocation - *Were processes used to protect against selection bias by concealing from the investigators how treatment was allocated at the start of the study?*

Check *"Yes"* if the investigator described his/her methods to protect treatment allocation. Minimum methodological standards for protecting treatment allocation include the use of sequentially numbered opaque, sealed envelopes (SNOSE); pharmacy controlled; numbered or coded containers; central randomisation—eg, by telephone to a trials office—or other method whose description contained elements convincing of concealment—eg, a secure computer-assisted method (Schulz et al. 1994; Schulz et al. 1995; Altman et al. 1990).

If the investigator states that concealment of allocation processes were used, but these minimum methodological standards are not met, then mark *"Partial"*.

If the investigator states that concealment of allocation processes were used, but does not describe the process that was actually used to protect treatment allocation, mark *"Partial"*.

If the investigator states that the study is randomized and makes no mention of concealment of allocation in the methodology, mark *"No"*.

If the study is not randomized, check *"No"* for concealment of allocation.

6. Blinding – *Were the investigators involved with performing the experiment, collecting data, and assessing the outcome of the experiment unaware of which subjects received the treatment and which did not?*

If authors state that they used blinded outcome assessment for an atherosclerotic related outcome, check "*Yes*". If blinding was not reported or the study was described as "open label", check "*No*".

7. Test Animal Characteristics and Environment

(a)Test Animal Description - *Did the authors sufficiently describe the test animal characteristics including the animal species, strain, sub-strain, genetic background, age, supplier, sex, weight?*

If investigators describe in detail the animal subjects, mark *"Yes"*. Sufficient detail should include the animal species, strain, sub-strain, genetic background, age, supplier, sex, weight. If four or more of these features are listed, mark *"Yes"*. If less than four of these features are listed (i.e. 1, 2, or 3 of these features are mentioned), check *"Partial"*. If none of these features are listed, mark *"No"*.

(b) Environmental Parameters - *Did the authors sufficiently describe the housing and husbandry, nutrition, water, temperature, lighting conditions?*

Sufficient detail means describing housing and husbandry, nutrition, water, temperature, and lighting conditions. If at least 3 of these characteristics are included, check "*Yes*". If less than 3 of these features are listed (i.e. 1 or 2 of these features are listed), check *"Partial"*. If none of these features are listed, mark "*No*".

8. Inclusion/Exclusion Criteria - *Were criteria used for including or excluding subjects specified?*

If inclusion and exclusion criteria are specified, with well-defined criteria appropriate to the study, mark *"Yes"*. Well-defined criteria should include specific information about the test subject such as animal disease state, age, sex, species, etc. If only the inclusion (exclusion) criteria is specified and not the exclusion (inclusion) criteria, check *"Partial."* If no information about inclusion and exclusion criteria is reported, mark *"No"*.

9. Dose / response model - *Was an appropriate dose-response model used given the research question and disease being modeled?*

If investigators used an appropriate dose / response model given the research question being asked, mark *"Yes"*. Authors should explain why they selected their dose/response model for this criterion to be met. If no justification for the dose/response model is provided, mark *"No"*. If authors use a single dose and justify their selection for that dose, mark *"Yes."* If authors use a single dose and do not justify their selection for that dose, mark *"No."*

10. All animals accounted for *- Did the investigator account for attrition bias by detailing when animals were removed from the study and for what reason they were removed?*

A change in the number of animal subjects in the study population may be determined from either the tables, text, or both. If the authors explain why the number of animals at the analytical stage is different from the beginning of the study, then mark "*Yes"*. If the authors only partially explain why the number of subjects changes, mark *"Partial"*.

Example: An example of a study coded as *"Partial"* would be one that starts off with 100 animals but during the analytical stage only has 50 remaining animals, and the author vaguely states that ½ of the animals were removed for developing a comorbidity, and makes no mention of important details including the timing of when the disease developed, the severity of the disease, or the cause of the disease.

If the authors do not explain why the number of animals at the analytical stage is different from the beginning of the study, then mark "*No"*.

If the authors do not report the number of animals in the analytical stage, then mark *"No"*.

11. Intention-to-Treat Analysis - *Did the authors perform an intention-to-treat analysis (ITT)?*
Mark "*Yes"* if the authors describe the protocol used for performing the ITT. Minimum requirements for an ITT is that animal subjects be kept in the same intervention groups to which they were randomized, regardless of the intervention they received (Cochrane Collaboration, 2008). If the authors report that ITT was performed, but do not describe the protocol, or if the above requirement is not met, mark *"Partial"*. If the authors make no mention of ITT, check "*No*". If the author state that "ITT will be performed" in the methods then mark "*Yes*". Mark "*No*" if the authors do not mention ITT. Also mark *"No"* if the authors mention in the methods that ITT was used, but in the results you can tell from the Ns that they clearly do not. Mark *"Partial"* if the authors report multiple analyses (some ITT, some not).

12. Optimal time window investigated - *Did the investigator provide sufficient time to pass before assessing the outcome? The optimal time window used in animal research should reflect the time needed to see the outcome.*

The optimal time window investigated will depend on the disease being studied. For this criterion to be met, the authors should define the time window and assess the outcome in a manner consistent with that time window. If the author defines the time window *a priori* and allows sufficient time to pass before assessing the outcome, then mark "*Yes"*. If the authors do not define a time window for the modeled disease or allow sufficient time to pass before assessing the outcome, then mark "*No"*. If authors do not justify their time window (i.e. they do not explain their time window), then mark "*No*". In a multiple outcomes study, if the authors only allow sufficient time or justify their time window for some outcomes, then mark *"Partial."*

13. Statement of Compliance with Animal Welfare Requirements – *Did the authors state whether or not they complied with regulatory requirements for the handling and treatment of test animals?*

Mark *"Yes"* if the authors state compliance with regulatory requirements. Mark *"No"* if the authors did not disclose whether or not they complied with regulatory requirements.

14. Sample size calculation - *Did the authors perform a sample size calculation to justify the total number of animals used in the study?*

If sample size calculations were performed *a priori*, check *"Yes"*. If sample size calculations were not performed, check *"No"*.

Part III. Results / Conclusions (double coded)

15. Results

Outcome Statistics
Record all summary statistics (exact units and values) that are presented in the results section for each selected outcome. Report the results of the statistical test and record the name of the test. Record whether the direction of the results favors the test statin, does not favor the test statin, or is neutral.

Things to consider when categorizing outcomes:

Code Individual Outcome Results as:
(1) Favorable if the result was statistically significant ($p < 0.05$) and in the direction of the statin being more efficacious or less harmful (in the case of adverse effects)

(2) Unfavorable if the result was not statistically significant ($p > 0.05$) or significant in the wrong direction (e.g., statin statistically more harmful than non-statin treatment group)

(3) Neutral if the statin was significantly different in the direction favoring the statin against one control group (e.g. early control) but not significantly different compared to a second control group (e.g., late control).

Example

Name of outcome	Result and Variance (eg, mean, SD; percent difference, CI)	Statistical test (result of test and test name)	Favors statin	Does not Favor statin	Neutral
% reduction in triglyceride concentration	20%, 11-34% CI	P = 0.003, t-test	X		

I. How to Code Results from Non-Equivalence Studies

A favorable result for a preclinical study is defined as a statistically significant "positive" result (for example, $p<0.05$) demonstrating efficacy of the statin OR a statistically significant result showing that the statin is less harmful than the comparator (less adverse effects).

Studies containing Active Comparators

- If statin is statistically less effective than the active comparator, but is statistically more effective than the control, mark "favors statin"
- If statin and active comparator are statistically equivalent but both are statistically more effective than the control, mark "favors statin".
- A neutral result is when it is right on the line of statistical significance or if we group outcomes as 3 favoring and 3 not favoring.

II. How to Code Results from Equivalence Studies

Code Individual Outcome Results as:
(1) Favorable if statin and active comparator are statistically equivalent but both the statin and active comparator are statistically more effective than the control. This supports the hypothesis that the statin is working the same as the comparator.

(2) Unfavorable if statin is statistically less effective than the active comparator, but both the statin and active comparator are statistically more effective than the control. This does not support the hypothesis that the statin is having the same effect as the comparator drug (even if it is different than control).

(3) Neutral if the statin, active comparator, and control are all the same (thus making it impossible to draw any conclusions).

16. Conclusion

Section 15 extracts data on the actual results of the study, whereas this part of the data extraction codes the conclusions of the study as stated by the authors. Note, the conclusions may not agree with the results, but we are coding the author's overall conclusion. This can usually be derived from the abstract and discussion sections.

Conclusions
Mark *"Favors statin"* if the overall conclusion suggests that the statin is efficacious and safe. If the authors hypothesize that the statin is efficacious and/or safe and this hypothesis is empirically supported, then mark *"Favors statin"*. For studies regarding combination therapies, if the conclusion supports the author's original hypothesis that the drugs have synergistic effects, this study is favorable towards the statin. Also, a conclusion can be favorable even if some limitations of the study are discussed.

Mark *"Does not favor statin"* if the overall conclusion suggests that the statin is not efficacious and safe and / or that the hypothesis of the paper was NOT supported. Also mark *"Does not favor statin"* if the overall conclusion states that the statin is less effective or less safe than the active comparator.

Mark *"Neutral"* conclusion if the paper does not draw a conclusion regarding the statin or states that the limitations of the study are so severe that the results are not valid. If neither the hypothesis nor the conclusion mentions the statin, the author does not draw a conclusion. Also mark *"Neutral"* for studies that state that the statin is comparable to the active comparator. For studies regarding combination therapies, if the conclusion supports that combination therapy is more effective than either therapy alone and makes no conclusive statement regarding monotherapy, then code this paper as "*Neutral"*.

17. Study Funding

(a): If the sponsor was involved in the study, mark *"sponsor involved".* If the authors "stated that sponsor was not involved in study design, data collection, analysis, etc", mark *"sponsor not involved".* If the sponsor was not mentioned, mark *"Not mentioned".* If the authors state there was no funder for their work, mark *"no funding".*

(b) If in 17a you marked "sponsor involved", list exactly what the sponsor did.

(c): If the study was industry-funded, mark, *"Industry".* If the study was not funded by industry, mark *"Non-industry".* If there was no funding statement, mark, *"no disclosure statement."* If the study was not funded, mark *"no funding."*

(d) List the name of the industry or non-industry source of funding, if mentioned.

18. Financial conflict of interest - *Did the investigator(s) disclose whether or not he/she has a financial conflict of interest?*

Mark *"Yes"* if any author discloses that he/she has a financial conflict of interest. If authors do not disclose whether or not they have a financial conflict of interest, check *"No disclosure statement"*. If all authors disclose that they have no financial conflicts of interest, *check "No"*. A financial conflict of interest consists of patents, stock, research funding, grants, gifts, consultancy, royalties, expert testimony, services on industry speaker's bureau, payments for manuscript preparation or review, travel, lectures, etc., or other relevant financial activities/relationships. This item pertains to the personal interests of the investigators and is coded separately from the funding source for the study.

References

Schulz KF, Chalmers I, Grimes DA, Altman DG. Assessing the quality of randomization from reports of controlled trials published inbobstetrics and gynecology journals. *JAMA* 1994; **272:** 125–28.

Schulz KF, Chalmers I, Hayes RJ, Altman DG. Empirical evidence of bias: dimensions of methodological quality associated with estimates of treatment effects in controlled trials. *JAMA* 1995; **273:** 408–12.

Altman DG, Doré CJ. Randomisation and baseline comparisons in clinical trials. *Lancet* 1990; **335:** 149–53.

Festing MFW, Altman DG (2002) Guidelines for the design and statistical analysis of experiments using laboratory animals. ILAR J 43: 244-258.

Cochrane-Collaboration, John Wiley & Sons L (2008) Cochrane Handbook for Systematic Reviews of Interventions; Higgins JP, Altman DG, editors. West Sussex, England: John Wiley & Sons Ltd, The Atrium Southern Gate, Chichester, West Sussex.

Appendix

Randomization. Describes whether or not treatment was randomly allocated to animal subjects so that each subject has an equal likelihood of receiving the intervention.

Concealment of Allocation. Describes whether or not processes were used to protect treatment allocation.

Blinding. Relates to whether or not the investigator involved with performing the experiment, collecting data, and assessing the outcome of the experiment was unaware of which subjects received the treatment and which did not.

Test animal descriptions. Describes the test animal characteristics including, the animal species, strain, sub-strain, genetic background, age, supplier, sex, weight. At least one of these characteristics must be present for this criterion to be met.

Environmental Parameters. Describes whether or not the authors described the housing and husbandry, nutrition, water, temperature, lighting conditions?

Inclusion/exclusion criteria. Describes the process used for including or excluding subjects.

Dose / response model. Describes whether or not an appropriate dose-response model was used given the research question and disease being modeled.

All animals accounted for. Describes whether or not the investigator accounts for attrition bias by detailing when animals were removed from the study and for what reason they were removed.

Intention-to-Treat Analysis. Describes whether or not the investigator performed an intention-to-treat analysis.

Optimal time window investigated. Describes whether or not the investigator provided sufficient time to pass before assessing the outcome. The optimal time window used in animal research should reflect the time needed to see the outcome.

Statement of Compliance with Animal Welfare Requirements. Describes whether or not the author(s) disclosed filling out a statement of compliance with regulatory requirements for the handling and treatment of test animals?

Sample size calculation. Describes how the total number of animals used in the study was determined.

Financial conflict of interest. This criterion is used to describe if the investigator(s) disclosed whether or not he/she has a financial conflict of interest.
